# Retinal Arterioles Narrow with Increasing Duration of Anti-Retroviral Therapy in HIV Infection: A Novel Estimator of Vascular Risk in HIV?

**DOI:** 10.1371/journal.pone.0051405

**Published:** 2012-12-10

**Authors:** Sophia Pathai, Helen A. Weiss, Stephen D. Lawn, Tunde Peto, Leris M. D’Costa, Colin Cook, Tien Y. Wong, Clare E. Gilbert

**Affiliations:** 1 International Centre for Eye Health, Department of Clinical Research, Faculty of Infectious and Tropical Diseases, London School of Hygiene & Tropical Medicine, London, United Kingdom; 2 Desmond Tutu HIV Centre, Institute of Infectious Diseases and Molecular Medicine, Faculty of Health Sciences, University of Cape Town, Cape Town, South Africa; 3 MRC Tropical Epidemiology Group, Faculty of Epidemiology and Population Health, London School of Hygiene & Tropical Medicine, London, United Kingdom; 4 Department of Clinical Research, Faculty of Infectious and Tropical Diseases, London School of Hygiene & Tropical Medicine, London, United Kingdom; 5 NIHR Biomedical Research Centre for Ophthalmology, at Moorfields Eye Hospital NHS Foundation Trust and UCL Institute of Ophthalmology, London, United Kingdom; 6 Department of Ophthalmology, Faculty of Health Sciences, University of Cape Town, Groote Schuur Hospital, Cape Town, South Africa; 7 Singapore Eye Research Institute, National University of Singapore, Singapore, Singapore; University of Texas Health Science Center San Antonio Texas, United States of America

## Abstract

**Objectives:**

HIV infection is associated with an increased risk of age-related morbidity mediated by immune dysfunction, atherosclerosis and inflammation. Changes in retinal vessel calibre may reflect cumulative structural damage arising from these mechanisms. The relationship of retinal vessel calibre with clinical and demographic characteristics was investigated in a population of HIV-infected individuals in South Africa.

**Methods:**

Case-control study of 491 adults ≥30 years, composed of 242 HIV-infected adults and 249 age- and gender-matched HIV-negative controls. Retinal vessel calibre was measured using computer-assisted techniques to determine mean arteriolar and venular diameters of each eye.

**Results:**

The median age was 40 years (IQR: 35–48 years). Among HIV-infected adults, 87.1% were receiving highly active antiretroviral therapy (HAART) (median duration, 58 months), their median CD4 count was 468 cells/µL, and 84.3% had undetectable plasma viral load. Unadjusted mean retinal arteriolar diameters were 163.67±17.69 µm in cases and 161.34±17.38 µm in controls (p = 0.15). Unadjusted mean venular diameters were 267.77±18.21 µm in cases and 270.81±18.98 µm in controls (p = 0.07). Age modified the effect of retinal arteriolar and venular diameters in relation to HIV status, with a tendency towards narrower retinal diameters in HIV cases but not in controls. Among cases, retinal arteriolar diameters narrowed with increasing duration of HAART, independently of age (167.83 µm <3 years of HAART vs. 158.89 µm >6 years, p-trend = 0.02), and with a HIV viral load >10,000 copies/mL while on HAART (p = 0.05). HIV-related venular changes were not detected.

**Conclusions:**

Narrowing of retinal arteriolar diameters is associated with HAART duration and viral load, and may reflect heightened inflammatory and pro-atherogenic states of the systemic vasculature. Measurement of retinal vascular calibre could be an innovative non-invasive method of estimating vascular risk in HIV-infected individuals.

## Introduction

HIV infection and highly active antiretroviral therapy (HAART) exacerbate a range of systemic age-related conditions such that HIV-infected patients are at increased risk of age-related non-AIDS-related morbidity and mortality compared with HIV-uninfected persons [1]–[3]. The emerging scenario is that of HIV population cohorts who are aging chronologically, but also likely to be undergoing accelerated physiological and immunological senescence. Mechanisms underlying accelerated aging include increased inflammation and immune dysfunction. The micro-vascular circulation may reflect cumulative structural damage arising from these processes. However, current methods to investigate the micro-circulation are invasive and require specialist expertise.

The retina represents a unique location where the micro-vasculature can be directly and non-invasively visualised. Validated and objective quantitative measurement of retinal vessel diameters is possible using semi-automated software applied to digitized retinal photographs [4]. This technique has been used in several population-based studies, and is established as a valid and efficient biomarker of systemic vascular disease [5]–[8]. Retinal vascular calibre is considered a structural marker of vascular pathology reflecting the interplay of systemic, environmental and genetic factors [9]. For example, changes in arteriolar calibre are strongly associated with chronological age, hypertension and cardiovascular disease, whereas venular calibre changes represent chronological age as well as inflammatory and cerebrovascular diseases. [10], [11]. Seemingly small reductions in retinal arteriolar calibre are associated with clinically relevant changes in blood pressure, e.g., a 10-mmHg increase in systolic BP is associated with a 1.1 µm reduction in arteriolar calibre [12]. A 20 µm increase in retinal venular calibre is associated with a coronary heart disease hazard ratio of 1.16 (95% CI: 1.06–1.26) in women.

There is limited information regarding relationships between retinal vessel calibre and HIV status, particularly in the context of premature aging and risk of age-related co-morbidities such as cardiovascular disease. Furthermore, data are lacking within sub-Saharan Africa, a region where the population of older HIV-infected persons is rapidly growing as individuals initiate HAART at increasingly early stages of the disease [13]. Assessment of retinal vessel calibre can provide a non-invasive and representative model to objectively assess changes in the micro-circulation in HIV infection and with HAART.

The objective of this study was to investigate the relationship of retinal vessel calibre with clinical and demographic characteristics in a cohort of HIV-infected individuals in South Africa in comparison with a matched population of uninfected individuals. We hypothesized that retinal vascular calibre would be altered in patients with HIV, who are known to have both elevated cardiovascular risk and chronic, systemic inflammation related to HIV-related accelerated aging [14], [15]. Cases and controls were recruited from neighbouring townships in Cape Town. The hypothesis of accelerated aging in HIV has received criticism primarily due to limitations in characterization of participants, and the possibility of differential exposure to potential risk factors (e.g. smoking, substance abuse) between HIV-infected and uninfected populations [16]. By recruiting from the same community, we aimed to reduce the likelihood of differential risk exposure in line with the recommendation for careful study design when investigating premature aging in HIV [17].

## Methods

### Ethics Statement

The study was approved by the London School of Hygiene and Tropical Medicine Ethics Committee and the University of Cape Town Faculty of Health Sciences Ethics Committee, and adhered to the tenets of the Declaration of Helsinki. Written informed consent was obtained from all participants.

### Study Participants

HIV-infected participants (cases) aged ≥30 years were enrolled from a community-based HIV treatment centre in Nyanga district of Cape Town [18], [19]. All participants had a confirmed serological diagnosis of HIV and were either about to commence HAART (HAART-naïve), or were already on first-line HAART.

A control group of HIV-uninfected participants was recruited using frequency-matching by gender and 5-year age categories. Controls were enrolled from participants confirmed to be HIV-negative attending an HIV prevention trials site (Emavundleni Centre), located within the same district as the HIV treatment centre. These two centres were chosen as individuals attending them were drawn from the same community and were therefore likely to have similar socio-demographic characteristics.

### Data Collection

Socio-demographic information and medical history were obtained by questioning participants in their first language (Xhosa or English). Clinical information was obtained from medical case notes where required. Co-morbidity was defined as the concurrent presence of one or more chronic diseases or conditions including cardiovascular disease, chronic renal failure, airways disease and malignancy (both AIDS and non-AIDS defining). Cardiovascular diseases included myocardial infarction and cerebrovascular disease. Blood pressure (BP) was measured with a digital sphygmomanometer with a cuff of appropriate size. Mean arterial blood pressure (MABP) was defined as two-thirds of the diastolic plus one-third of the systolic BP. Hypertension was defined as a systolic BP of 140mmHg or higher, diastolic BP of 90mmHg or higher, or the combination of self-reported high BP diagnosis and the use of anti-hypertensive medications [20]. Body mass index (BMI) was defined as weight (in kilograms)/height^2^. HIV infection characteristics including duration of HAART, type of HAART regimen, nadir and current CD4 count and viral load (VL) were available from the clinic database.

### Retinal Vessel Measurement

**Figure 1:**
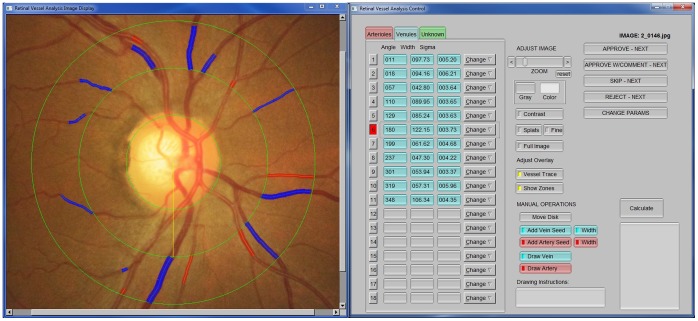
Retinal vessel grading assessment. Right: fundus with automated measurements (red = arterioles, blue = venules). Left: venular diameters output.

All participants had stereoscopic 30 degree colour retinal photographs taken of both eyes under pharmacological pupil dilation with a fundus camera (model CF-2; Canon Inc., Tokyo, Japan). Images were centred on the optic disc. Vessel calibre indices were determined in a semi-automated manner using the IVAN computer program (Singapore Eye Research Institute, Singapore) using a standardized protocol described previously [4]. In summary, the 6 largest arterioles and venules in a ring-shaped area located between 0.5 and 1.0 disc diameter from the optic disc margin were identified (Figure 1). Computer software measured the calibre of these individual vessels, then combined them into 2 summary variables for the eye: the projected calibre size of the central retinal artery (central retinal artery equivalent [CRAE]), and the projected calibre size of the central retinal vein (central retinal vein equivalent [CRVE]), using formulas derived by Parr and Spears [21], [22] and Hubbard [23], with revision by Knudtson [24]. A retinal photograph was considered ungradable if eyes had <4 acceptable measurements of either vessel type. The inter-grader and intra-grader grading reliabilities were assessed using a random subsample of 100 photographs reviewed four weeks after the initial grading. The intra- and inter-grader intraclass correlation coefficients ranged from 0.71 to 0.93.

### Data Analysis and Statistical Methods

If gradable images were available for both eyes, one eye was randomly selected for vessel calibre assessment as good correlation between eyes has been demonstrated using this software, and is considered sufficient for assessing relationships to systemic health status [25]. If there was only one gradable image, this one was used, and if neither eye had a gradable image the participant was excluded. Retinal vessel data (CRAE and CRVE) were analyzed as continuous variables. Univariable linear regression was performed to compare mean retinal arteriolar and venular diameters respectively by gender and HIV status (no; yes). Multivariable linear regression models were used to examine the relationships of retinal vessel diameters as the dependent variable with HIV status and explanatory variables (age group (30–39; 40–49; >50 years), gender, mean arterial blood pressure; BMI, smoking and diastolic blood pressure). Models included an interaction term between HIV and each explanatory variable. Marginal adjusted means for retinal vessel diameters were estimated at the mean value of covariates in the model. The Wald test was used to assess statistical significance of the interaction of HIV status with each explanatory variable on retinal vessel diameter. Retinal arteriolar and venular calibre are highly correlated, and to account for potential confounding we adjusted for the fellow vessel in multivariable analyses (i.e. adjustment for arteriolar calibre in analyses of venular calibre and vice versa) [26]. All analyses were performed with Stata 12 (Stata Corp, USA).

## Results

### Participant Characteristics

491 participants were evaluated, of whom 242 had HIV-infection, and 249 were uninfected controls. The mean age of the HIV-infected population was 41.2±0.5 years, and 42.5±0.6 years in the uninfected group (p = 0.10). Cases had lower BMI, were less likely to be smokers, and more likely to have current or previous TB than controls (Table 1). Overall, 72.7% of cases had a history of WHO stage 3 or stage 4 defining illness. The current CD4 count among participants receiving HAART was 468 cells/µL (interquartile range [IQR], 325–607 cells/µL) and 84.0% had undetectable VL. Median treatment duration on HAART was 58 months (IQR: 34–75 months). 12.0% were HAART naïve, and had correspondingly lower CD4 counts and higher VL.

**Table 1:** Demographic characteristics of study population.

Variable	Cases (242) % (n)	Controls (249) % (n)	P-value
Age (mean±SD)	41.2±0.5	42.5±0.6	0.10
Age (years) by group			
30–39	49.2 (119)	47.0 (117)	0.54
40–49	33.5 (81)	31.7 (79)	
>50	17.3 (42)	21.3 (53)	
Male gender	25.6 (62)	24.1 (60)	0.70
Hypertension status			
Hypertensive	27.3 (66)	29.3 (73)	0.62
Mean arterial blood pressure	94.37±14.64	95.53±13.90	0.37
BMI (kg/m^2^)	27.8±6.5	31.5±8.8	<0.0001
Co-morbidity			
None	71.1 (172)	69.5 (173)	
One or more	28.9 (70)	30.5 (76)	0.70
Smoking status			
Smoker	15.3 (37)	27.3 (68)	0.001
TB status			
No history	30.6 (74)	88.0 (219)	<0.0001
Current	4.1 (10)	0.4 (1)	
Previous	64.3 (158)	11.7 (29)	
**HIV characteristics (n = 242) (%) n or median (IQR)**			
WHO stage			
½	27.3 (66)		
3/4	72.7 (176)		
HAART naïve	12.0 (29)		
CD4 count in HAART naïve group (n = 29)	170 (84–201)		
Log_10_VL* in HAART naïve group (n = 19)	4.81 (4.11–5.14)		
Current CD4 count in HAART group	468 (325–607)		
Nadir CD4 count in HAART group	127 (76–171)		
% with detectable VL in HAART group	16.0 (34)		
Peak Log_10_VL in HAART group	4.56 (3.84–4.98)		
Duration of HAART, months	58 (34–75)		
HAART Regimen			
NNRTI-based*	59.6 (127)		
Other	40.4 (86)		

* VL = HIV RNA Viral load; NNRTI - non-nucleoside reverse transcriptase inhibitor;

### Retinal Photography and Vessel Grading

All participants underwent retinal photography. Photographs were ungradable for retinal vessel diameters in 12 individuals (2.4%), who were older than those with gradable photographs (p = 0.001) but otherwise had similar characteristics (p>0.05 for all characteristics; data not shown). The proportion of cases and controls with ungradable photos was similar (2.48% vs. 2.41%; p = 0.96).

### Overall Retinal Vessel Measurements

Measurements are summarised in Table 2. The following comparisons were not statistically different: arteriole diameters between cases and controls, overall (p = 0.15) nor by gender (p = 0.68 for males, p = 0.14 for females). However, venules were narrower in cases than controls although this did not reach statistical significance (p = 0.07). Male cases had significantly narrower venules (265.72±18.04 µm) than male controls (274.55±21.66 µm; p = 0.02) but this association was not seen in females (p = 0.55).

**Table 2:** Retinal vessel calibre in study population.

	Mean arteriolar diameter			Mean venular diameter		
	HIV+ cases	Uninfected controls	P-value	HIV+ cases	Uninfected controls	P-value
**Overall**	163.67±17.69	161.34±17.38	0.15	267.77±18.21	270.81±18.98	0.07
**Males**	162.00±17.68	160.53±20.48	0.68	265.72±18.04	274.55±21.66	0.02
**Females**	164.24±17.71	161.60±16.33	0.14	268.47±18.27	269.62±17.94	0.55

Measurements are in µm and represent mean ± standard deviation.

### Retinal Vessel Calibre and Association with HIV and Age

In unadjusted analyses, there was a significant trend of narrower arteriolar diameters (P-trend = 0.002), and venular diameters (P-trend = 0.001) with increasing age in the HIV-infected group, also evident when stratified by gender (data not shown). This trend was not seen consistently in the control group. Table 3 reports adjusted mean vascular diameters by HIV status, stratified by other covariates. In relation to mean arteriolar diameters, age and hypertension status modified the association in relation to HIV status (P values for interaction 0.01). Mean arteriolar diameters tended to decrease with age among HIV-infected cases, and to increase among controls. Among controls, those with hypertension had wider arteriolar diameters than those without hypertension (167.97 vs. 158.28 µm; p = 0.002), whereas among HIV cases there was little association with hypertension (164.82 vs. 163.53 µm; p = 0.69). Mean venular diameter also decreased with age among HIV cases, but there was no association among controls (p-interaction = 0.07). In addition, venular diameter tended to be greater among males than females, and smokers than non-smokers among controls, but little difference was seen among cases.

**Table 3:** Retinal Arteriolar and Venular Diameters in Relationship to HIV status, Stratified by Potential Effect Modifiers*.

	Mean arteriolar diameter µm (SE)			Mean venular diameter µm (SE)		
	HIV-infected (n = 236)	Controls (n = 243)	P-value for interaction	HIV-infected(n = 236)	Controls (n = 243)	P-value for interaction
Overall	163.95 (1.14)	161.08 (1.12)		268.17 (1.22)	270.43 (1.20)	
Age group						
30–39	165.86 (1.64)	161.27 (1.60)	0.01	271.34 (1.77)	269.49 (1.73)	0.07
40–49	163.47 (1.93)	157.94 (1.94)		265.59 (2.07)	271.43 (2.10)	
>50	159.13 (2.72)	166.24 (2.55)		264.45 (2.92)	270.96 (2.75)	
*P-value*	*0.11*	*0.03*		*0.05*	*0.76*	
Sex						
Male	161.02 (2.41)	158.29 (2.56)	0.22	266.68 (2.56)	276.13 (2.72)	0.02
Female	164.92 (1.34)	162.00 (1.36)		268.73 (1.44)	268.54 (1.44)	
*P-value*	*0.17*	*0.23*		*0.50*	*0.02*	
Cigarette use						
No	163.73 (1.24)	160.21 (1.34)	0.16	268.46 (1.32)	268.69 (1.44)	0.03
Yes	164.20 (3.09)	163.93 (2.25)		265.47 (3.29)	275.69 (2.38)	
*P-value*	*0.89*	*0.17*		*0.41*	*0.01*	
Hypertension status						
No	163.53 (1.45)	158.28 (1.42)	0.01	270.21 (1.57)	271.86 (1.54)	0.38
Yes	164.82 (2.60)	167.97 (2.46)		262.94 (2.78)	266.70 (2.66)	
*P-value*	*0.69*	*0.002*		*0.04*	*0.12*	
BMI						
<20	166.73 (3.71)	166.06 (4.36)	0.13	262.20 (3.96)	274.52 (4.66)	0.77
20–24.9	166.48 (2.20)	159.24 (2.75)		266.10 (2.36)	267.22 (2.94)	
25–29.9	160.77 (2.04)	160.77 (2.43)		266.56 (2.19)	270.04 (2.60)	
>30	163.95 (2.00)	161.18 (1.55)		271.78 (2.14)	271.93 (1.66)	
*P-value*	*0.24*	*0.59*		*0.11*	*0.41*	

Adjusted for gender, age, hypertension status, smoking status, BMI, diastolic BP and associate vessel diameter (i.e. CRVE for CRAE analyses, and CRAE for CRVE analyses), categorised as shown in the Table.

CRAE/CRVE = Central retinal artery equivalent/central retinal vein equivalent.

### Retinal Vessel Calibre in HIV-infected Participants on HAART

We investigated the associations between mean arteriolar and venular diameter and clinical/demographic factors in participants on HAART (Table 4). There was a trend of increasing age being associated with narrowing of retinal arterioles (P-trend = 0.08). A longer duration of HAART was associated with narrowing of arterioles. Mean arteriolar diameter ranged from 167.83 µm in those with less than 3 years duration of HAART to 158.89 µm with more than 6 years of treatment (P-trend = 0.02). A higher current viral load while on HAART (>10,000 copies/ml) was associated with narrower arterioles (p = 0.05). There was an association of narrowed arterioles with hypertension (p = 0.03) in this group, however this association was not apparent after adjustment for HIV-related factors (data not shown). Venular diameters narrowed with increasing age (P-trend = 0.02). A higher current viral load was associated with wider venular diameters in the unadjusted estimates (266.36 µm for VL <10, 000 copies/ml vs. 279.10 µm for VL>10,000 copies/ml (p = 0.02). The trend of wider venules with higher viral load was apparent in the fully adjusted model, but did not reach statistical significance (p = 0.20).

**Table 4:** Retinal vessel diameters in association to HIV-related factors in participants on HAART.

		Arteriolar diameter (n = 207)		Venular diameter (n = 207)	
	N	Mean (SE), µm	*P*	Mean (SE), µm	*P*
Age group (years)					
30–39	99	164.81 (1.87)		269.93 (1.91)	
40–49	73	163.40 (2.17)	0.08*	266.49 (2.21)	0.02*
>50	35	157.31(3.20)		260.37 (3.26)	
Sex					
Male	50	160.67 (3.05)		265.78 (3.12)	
Female	157	163.80 (1.49)	0.37	267.52 (1.53)	0.64
Hypertension status					
No	148	161.58 (1.93)		268.31 (1.98)	
Yes	59	166.72 (4.00)	0.35	264.06 (4.08)	0.42
HAART duration, months					
0–36	60	167.83 (2.56)		269.70 (2.63)	
37–72	86	162.65 (1.97)		265.91 (2.01)	
>73	61	158.89 (2.38)	0.02*	266.22 (2.45)	0. 57
Current CD4 count					
<500 cells/µL	120	163.04 (1.66)		266.80 (1.70)	
>501 cells/µL	87	163.05 (1.99)	0.98	267.51 (2.03)	0.78
Nadir CD4 count					
<200 cells/µL	184	162.85 (1.29)		266.37 (1.31)	
>201 cells/µL	23	164.58 (3.94)	0.67	272.92 (4.01)	0.13
Current HIV viral load					
<10,000 copies/ml	195	163.71 (1.25)		266.65 (1.27)	
>10,000 copies/ml	12	152.21 (5.64)	0.05	274.40 (5.79)	0.20
Peak HIV viral load					
<10,000 copies/ml	59	164.32 (2.37)		268.59 (2.42)	
>10,000 copies/ml	148	162.54 (1.45)	0.50	266.51 (1.48)	0.46

Adjusted for age, gender, hypertension status, mean arterial blood pressure (MABP), smoking status, BMI, diastolic BP, associate vessel diameter (i.e. CRVE for CRAE analyses, and CRAE for CRVE analyses), current CD4 count and nadir CD4 count, current VL and peak VL, HAART duration, HAART regimen, WHO clinical stage (1/2 or 3/4) and TB status (current or past history vs. no history).

* P-value for test of trend.

## Discussion

We assessed retinal vessel calibre in HIV-infected individuals in South Africa. This study provides clear evidence that retinal arteriolar diameters narrow with increasing duration of HAART and with higher HIV viral load, independently of age. The excess age-related morbidity demonstrated in HIV-infected individuals has a significant vascular component including cardiovascular, renal and cerebrovascular disease, and retinal vessel calibre measurement has been demonstrated as a strong biomarker of systemic vascular disease.

We found an 8.9 µm decrease in arteriolar diameter in participants who had been on HAART for >6 years after adjustment for age and HIV-related factors. Our findings are in alignment with data from the Longitudinal Studies of the Ocular Complications of AIDS (LSOCA) [27], in particular, the association of narrower retinal arteriolar diameter with exposure to HAART. In our study population we additionally demonstrated that retinal arteriolar diameter decreases with increasing duration of HAART. The association of retinal arteriolar calibre with cardiovascular risk is well documented. Retinal arteriolar diameter (CRAE) is independently associated with increased carotid intima thickness [12], and with higher cardiovascular mortality risk in older persons [5], [28]. An important cause of premature morbidity and mortality in HIV-infected individuals is from cardiovascular complications [29]. HAART-treated patients have a greater prevalence of atherosclerosis and vascular dysfunction than age-matched uninfected adults [30]. Enhanced endothelial dysfunction (measured by flow-mediated dilation) and increased carotid intima media thickness have been demonstrated in HIV cases compared to controls, despite antiretroviral therapy and adjustment for traditional CVD risk factors [31]–[35]. It is plausible that narrower retinal arteriolar diameter is related to excess cardiovascular risk in patients on HAART. The risk of cardiovascular disease appears to be greatest with protease inhibitors (PIs) compared to non-nucleoside reverse transcriptase inhibitors (NNRTIs) [36], [37]. The majority of our study population were treated with NNRTIs, and we postulate that the magnitude of arteriolar narrowing might be greater in populations where treatment is PI-based.

Higher viral load while on HAART (>10,000 copies/mL) was associated with wider venular calibre in unadjusted estimates; this trend remained in an adjusted model, but did not achieve statistical significance. Endothelial dysfunction is associated with larger retinal venules independent of traditional cardiovascular risk factors [38]. Larger venular calibre is also associated with higher levels of inflammatory markers such as interleukin-6 [39], [40]. We postulate that in our HIV-infected population higher HIV viral loads may cause inflammation and/or endothelial dysfunction manifest as retinal venular dilation. The association of *narrow* venular diameter with increasing age was stronger in an adjusted model. This may reflect lack of statistical power, or possibly that the ‘aging’ phenotype plays a larger role in determining venular calibre in HIV. Narrow arteriolar calibre is also associated with inflammation in diabetes possibly explaining the finding of narrower CRAE with higher viral load [11].

The strong association between increasing age and narrowed retinal vessels has been demonstrated in several study populations [41]–[43]. This association was demonstrated in our cohort of HIV-infected individuals, and in a US HIV population [27]. We hypothesized that retinal vascular changes typically occurring in older populations might occur earlier in life in HIV-infected individuals. For venular diameters this appears to be consistent, however, this trend was not apparent in retinal arterioles. The interaction of age with HIV status in determining retinal arteriolar diameter is novel and biologically difficult to explain. Graders were masked to the HIV status of the participants, and mis-grading would have caused random misclassification. There may be residual confounding and bias accounting for this finding, or this scenario could have occurred by chance. The effect of age in relation to HIV status, and possible effect modification warrants further investigation.

The strengths of this study include the high proportion of gradable digital retinal photographs, and use of a well-established, standardized computer-based technique to measure retinal vascular calibre. The study design also permitted inclusion of an age/gender matched control group with a similar socio-demographic profile as the cases. The hypothesis of accelerated aging in HIV has received criticism primarily due to limitations in characterization of participants, and the possibility of differential exposure to potential risk factors between HIV-infected and uninfected populations [16], [44], [45]. By recruiting from the same community, we aimed to reduce the likelihood of differential risk exposure.

Despite overall high reproducibility using computer-assisted methods, many factors may affect vessel measurement, some of which are inherent. For example, slight changes in vessel diameter with the cardiac cycle (due to pulsatility) may result in variation in vessel measurements [46] but fluctuations are small and random [9], causing non-differential misclassification. Measurement of vessel diameters from colour retinal photographs may underestimate true vascular width because only the red blood cell column is measured, and not the peripheral plasma cuff. Other factors relate to the population studied e.g. clarity of the ocular media and hence photographs, and the presence of greater retinal pigment, as in African retinas, may overestimate retinal vessel diameter [47]. Our study design does not permit conclusions about the temporal relationship between changes in retinal calibre and subsequent risk of morbidity (e.g. cardiovascular events) or mortality from these data. In addition, we cannot make any inference about whether HIV infection or HAART is primarily responsible for the changes in vascular calibre due to the low proportion of HAART-naïve individuals recruited. Finally, as both case and control populations are of African ancestry these findings may not be generalisable to other ethnic groups.

We present novel data on retinal vascular calibre in HIV. It is reassuring that our findings are in alignment with a US HIV cohort [27]. However, compared to US HIV populations, it is likely that African HIV populations will have experienced a shorter duration of HAART and initiated treatment at lower CD4 counts. The atherogenic, inflammatory and aging effects of HIV and HAART and related changes in retinal vascular calibre may thus follow a different trajectory, with longitudinal data needed to assess this definitively. It is also unclear whether HIV infection or treatment with HAART is primarily responsible for changes in vascular calibre. In addition, epigenetic and genetic variation may contribute to an individual’s susceptibility to non-HIV age-related morbidity and to vascular structural changes.

The challenge of managing excess age-related morbidity in HIV-infected individuals will increase as HIV populations live longer and HAART coverage expands. Stratification of vascular risk among HIV-infected individuals, particularly cardiovascular risk, and implementing preventive strategies will be important priorities for patient management. Cardiovascular risk assessment tools already exist such as the Framingham Risk Scores [48], however, specific tools tailored to HIV-infected populations are necessary [49]. Measurement of retinal vessel calibre is validated, non-invasive, and can be performed quickly by non-clinical personnel. Our data support the hypothesis that retinal vascular calibre changes occur in HIV infection, reflecting systemic vascular pathology. Longitudinal studies are needed to confirm this hypothesis as well as validation studies to explore the role of retinal vessel measurement as a tool in HIV-related vascular risk estimation.
